# Characterization of three pyranose dehydrogenase isoforms from the litter-decomposing basidiomycete *Leucoagaricus meleagris* (syn. *Agaricus meleagris*)

**DOI:** 10.1007/s00253-016-8051-1

**Published:** 2016-12-19

**Authors:** Michael M. H. Graf, Sandra Weber, Daniel Kracher, Roman Kittl, Christoph Sygmund, Roland Ludwig, Clemens Peterbauer, Dietmar Haltrich

**Affiliations:** 1 Food Biotechnology Laboratory, Department of Food Science and Technology, University of Natural Resources and Life Sciences (BOKU), Muthgasse 18, 1190 Vienna, Austria; 2 BioToP—The Doctoral Programme on Biomolecular Technology of Proteins, Muthgasse 18, 1190 Vienna, Austria

**Keywords:** *Agaricus meleagris*, Pyranose dehydrogenase, Flavoprotein, Enzyme isoform, Enzyme multiplicity, 6-Hydroxy-FAD

## Abstract

Multigenicity is commonly found in fungal enzyme systems, with the purpose of functional compensation upon deficiency of one of its members or leading to enzyme isoforms with new functionalities through gene diversification. Three genes of the flavin-dependent glucose–methanol–choline (GMC) oxidoreductase pyranose dehydrogenase (*Am*PDH) were previously identified in the litter-degrading fungus *Agaricus* (*Leucoagaricus*) *meleagris*, of which only *Am*PDH1 was successfully expressed and characterized. The aim of this work was to study the biophysical and biochemical properties of *Am*PDH2 and *Am*PDH3 and compare them with those of *Am*PDH1. *Am*PDH1, *Am*PDH2 and *Am*PDH3 showed negligible oxygen reactivity and possess a covalently tethered FAD cofactor. All three isoforms can oxidise a range of different monosaccarides and oligosaccharides including glucose, mannose, galactose and xylose, which are the main constituent sugars of cellulose and hemicelluloses, and judging from the apparent steady-state kinetics determined for these sugars, the three isoforms do not show significant differences pertaining to their reaction with sugar substrates. They oxidize glucose both at C2 and C3 and upon prolonged reaction C2 and C3 double-oxidized glucose is obtained, confirming that the *A. meleagris* genes *pdh2* (AY753308.1) and *pdh3* (DQ117577.1) indeed encode CAZy class AA3_2 pyranose dehydrogenases. While reactivity with electron donor substrates was comparable for the three *Am*PDH isoforms, their kinetic properties differed significantly for the model electron acceptor substrates tested, a radical (the 2,2′-azino-bis[3-ethylbenzothiazoline-6-sulphonic acid] cation radical), a quinone (benzoquinone) and a complexed iron ion (the ferricenium ion). Thus, a possible explanation for this PDH multiplicity in *A. meleagris* could be that different isoforms react preferentially with structurally different electron acceptors in vivo.

**Electronic supplementary material:**

The online version of this article (doi:10.1007/s00253-016-8051-1) contains supplementary material, which is available to authorized users.

## Introduction

Multigenicity is a feature commonly found in fungal enzyme systems. This is often caused by several paralogous genes present in an organism as a result of gene duplication events (Taylor 2011), with the purpose of functional compensation upon deficiencies of one of its members (Salame et al. 2013). Moreover, gene duplication events are often followed by gene diversification, this process being one of the most important mechanisms leading to enzyme isoforms with new functionalities (Force et al. 1999; Kilaru et al. 2006; Ohta 1991; Ramos et al. 2011). Examples of multigenicity in wood-decaying fungi include, e.g. the xenome, the protein machinery that detects, transports and metabolizes xenobiotics (Edwards et al. 2005; Morel et al. 2013) or various auxiliary redox enzymes that were added recently to the CAZy database (Levasseur et al. 2013). The xenome is constituted of multigenic, intracellular superfamilies of cytochrome P450 monooxygenases and glutathione transferases (Kües 2015). These superfamilies are especially prevalent in wood- and litter-degrading fungi, which often have to deal with harmful compounds derived from organic matter degradation, secondary metabolism of antagonists or human activities (Mathieu et al. 2013; Syed et al. 2014). The new class of auxiliary redox enzymes (auxiliary activities (AA)) added to the CAZy database comprises a range of oxidoreductases that are linked to lignocellulose breakdown. With respect to multigenicity, one of these auxiliary activities, lytic polysaccharide monooxygenase (LPMO, AA9), is noteworthy, with, for example, 30 and 11 candidate proteins in the genomes of the litter-degrading basidiomycetes *Coprinopsis cinerea* and *Agaricus bisporus*, respectively (Kracher et al. 2016; Morin et al. 2012; Stajich et al. 2010).

The plant litter-decomposing fungus *Agaricus meleagris* (synonyms: *Leucoagaricus meleagris*, *Agaricus moelleri*, *Agaricus praeclaresquamosus*, *Leucocoprinus meleagris* and *Lepiota meleagris*; in accordance with our previous publications, we here use the name *A. meleagris*) is found in temperate zones of the northern hemisphere, growing in mixed forests. Three pyranose dehydrogenase (*Am*PDH) encoding genes were previously isolated from *A. meleagris* strain CCBAS 907 (Culture Collection of Basidiomycetes of the Academy of Sciences; Prague, Czech Republic) and designated as *pdh1*, *pdh2* and *pdh3* (Kittl et al. 2008). Only the *Am*PDH1 protein has been successfully produced and characterized to date (Graf et al. 2013, 2014, 2015; Krondorfer et al. 2014a, b; Sedmera et al. 2006; Sygmund et al. 2008, 2012; Tan et al. 2013), and no evidence for the presence of *Am*PDH2 and *Am*PDH3 in culture supernatants of *A. meleagris* have been found so far. Glycosylated, monomeric *Am*PDH1 has a mass of approximately 75 kDa, contains a monocovalently tethered FAD cofactor and is a member of the glucose–methanol–choline (GMC) superfamily of oxidoreductases (Cavener 1992; Peterbauer and Volc 2010). In the CAZy databank (http://www.cazy.org), PDH (EC 1.1.99.29) is grouped in the auxiliary activity family 3 subfamily 2 (AA3_2). PDH activity was only detected in culture filtrates of several litter-decomposing basidiomycetes belonging to the *Agaricaceae* and *Lycoperdaceae*, but not in white-rot wood-decaying basidiomycetes (Volc et al. 2001). *Am*PDH1 is able to oxidize a large variety of carbohydrates occurring during lignocellulose degradation (Graf et al. 2014, 2015; Peterbauer and Volc 2010; Sedmera et al. 2006; Tan et al. 2013). These properties make the enzyme interesting for bioelectrochemical applications as well as for providing carbohydrate building blocks for organic synthesis (Peterbauer and Volc 2010; Yakovleva et al. 2015).

Although *Am*PDH2 and *Am*PDH3 have, to date, neither been isolated from its fungal source nor expressed and characterized, they most likely possess related properties to *Am*PDH1, since they share close similarities (Kittl et al. 2008): the mRNAs consist of 1809 bp translating into 602 amino acids for *pdh1* as well as 1803 bp translating into 600 amino acids for *pdh2* and *pdh3*. The three proteins have a large number of identical and conserved amino acids, 75 and 85% for *Am*PDH1 and *Am*PDH2, 76 and 85% for *Am*PDH1 and *Am*PDH3 and 84 and 92% for *Am*PDH2 and *Am*PDH3, respectively. A multiple sequence alignment of the three isoforms with Clustal Omega (Sievers et al. 2011) demonstrates that they share 409 chemically identical, 76 highly similar and 29 weakly similar residues (Supplementary Fig. S1). The close relationship of the three *Am*PDH isoforms allows the construction of homology models for *Am*PDH2 and *Am*PDH3 with high reliability, based on the 1.6-Å X-ray structure of *Am*PDH1 (Tan et al. 2013) (Supplementary Fig. S2). Those homology models suggest very similar active site architectures for the three enzymes, with one amino acid as the only exception. Val-511 in *Am*PDH1 is replaced by leucine (Leu-509) in *Am*PDH2 and by tryptophan in *Am*PDH3 (Trp-509). Whereas leucine is chemically and sterically similar to valine, tryptophan is much bulkier and when positioned in the active site might result in altered properties.

The aim of this study was to establish an expression and purification strategy for *Am*PDH2 and *Am*PDH3 and to biophysically and biochemically characterize these two proteins. Subsequently, the properties of all three isoforms were compared with the ultimate goal of better understanding *pdh* multigenicity in *A. meleagris.*

## Materials and methods

### Chemicals and vectors

All chemicals were of the highest purity available and bought from Sigma-Aldrich (St. Louis, MO, USA), VWR (Radnor, PA, USA) and Roth (Karlsruhe, Germany). Primers were from LGC Genomics (Teddington, UK) or Sigma-Aldrich. Restriction endonucleases, T4 DNA ligase and Phusion polymerase were from Thermo Fisher Scientific Biosciences (St. Leon-Rot, Germany). GoTaq polymerase was purchased from Promega (Madison, WI, USA). Zeocin and the pPICZB vector were obtained from Invitrogen (Carlsbad, CA, USA).

### Strains and media

Strains and media used for this study were essentially the same as previously reported (Graf et al. 2015; Krondorfer et al. 2014b). In short, *Escherichia coli* strain NEB5α was obtained from New England Biolabs (Ipswich, MA, USA) and *Pichia pastoris* strain X33 from Invitrogen. For fermenter cultivations, the basal salts medium with 4.35 mL/L PTM_1_ trace salts was used, which is described in detail by Invitrogen or in Krondorfer et al. (2014b).

### Plasmid construction for expression in *P. pastoris*

By using the primer pair pPICZB-6His-fw and pPICZB-6His-*Xba*I-rv (see Supplementary Table S1), the *myc*-epitope of the original pPICZB plasmid from Invitrogen was removed using the *Dpn*I-method as described in Graf et al. (2015), creating pPICZB-His_6_. The *A. meleagris pdh1* (*ampdh1*, GenBank accession number AY753306.1) gene in the pPICZB vector (Krondorfer et al. 2014b) was amplified with the primer pair *Am*PDH1-*Not*I-fw and *Am*PDH1-*Xba*I-rv (Supplementary Table S1), introducing a 5′-*Not*I and a 3′-*Xba*I restriction site. The purified product was digested with the endonucleases *Not*I and *Xba*I and ligated into the equally treated pPICZB-His_6_ vector, yielding pPICZB-His_6_-*Am*PDH1. The *A. meleagris pdh2* (*ampdh2*, AY753308.1) and *pdh3* (*ampdh3*, DQ117577.1) genes reported by Kittl et al. (2008) were amplified with the primer pair *Am*PDH2-*BstB*I-fw and *Am*PDH2-*Xba*I-rv or *Am*PDH3-*BstB*I-fw and *Am*PDH3-*Xba*I-rv (Supplementary Table S1), thereby introducing a 5′-*BstB*I and a 3′-*Xba*I restriction site, respectively. The purified products were digested with the endonucleases *BstB*I and *Xba*I and ligated into the equally treated pPICZB-His_6_ vector, yielding pPICZB-His_6_-*Am*PDH2 and pPICZB-His_6_-*Am*PDH3, respectively. The three constructs were subsequently propagated as described previously (Graf et al. 2015).

### Gene expression and protein purification

*Am*PDH1, *Am*PDH2 and *Am*PDH3 were essentially produced in cultivations in a 7-L laboratory fermenter as described for *Am*PDH1 (Graf et al. 2015), using basal salts fermentation medium. A purification scheme as described previously (Graf et al. 2015) was used. Briefly, this scheme is based on hydrophobic interaction chromatography (HIC) using a Phenyl Sepharose Fast Flow column (GE Healthcare, Little Chalfont, UK), equilibrated with 50 mM potassium phosphate buffer (pH 6.5, 40% saturation (NH_4_)_2_SO_4_), and immobilized metal affinity chromatography (IMAC) with a Ni^2+^-charged Chelating Sepharose Fast Flow column (GE Healthcare), equilibrated with 100 mM potassium phosphate buffer pH 7.0, 1 M NaCl and 5 mM imidazole. An additional ion exchange chromatography (IEX) step using a DEAE-Sepharose Fast Flow column (GE Healthcare) equilibrated with 100 mM potassium phosphate buffer pH 7.0 was added for *Am*PDH3 as a polishing step.

### Standard enzyme activity assay

Pyranose dehydrogenase (EC 1.1.99.29; pyranose/acceptor oxidoreductase) activity was assayed spectrophotometrically by following the glucose-dependent reduction of the ferrocenium cation (FC^+^; ferrocenium hexa-fluorophosphate (FcPF_6_)) to ferrocene at 300 nm (ε_300_ = 4.3 mM^−1^ cm^−1^) in 50 mmol Na phosphate buffer pH 7.5 for 3 min at 30 °C (Graf et al. 2015; Volc et al. 2001). One unit of PDH activity was defined as the amount of enzyme necessary for the reduction of 2 μmol of ferricenium ion per minute under the conditions described above.

The pH dependence of *Am*PDH activity with the 2,2′-azino-bis(3-ethylbenzothiazoline-6-sulphonic acid) cation radical (ABTS^·+^, a 1-e^−^ acceptor; measured at 414 nm, ε_414_ = 36 mM^−1^ cm^−1^), 1,4-benzoquinone (BQ, a 2-e^−^ acceptor; measured at 290 nm, ε_290_ = 2.3 mM^−1^ cm^−1^) and the ferrocenium ion (FC^+^, a 1-e^−^ acceptor) as electron acceptors was determined with an adapted standard enzyme activity assay, for which 40 mM Britton–Robinson buffer titrated to the desired pH value was used. The temperature dependence of *Am*PDH activity was probed with the standard enzyme activity assay at the indicated temperature.

### Molecular properties

Protein concentrations were determined according to Bradford with a pre-fabricated assay (BioRad, Hercules, CA, USA). SDS-PAGE and enzymatic deglycosylation with PNGase F were conducted as described previously (Krondorfer et al. 2014b; Sygmund et al. 2008). Far-UV electronic circular dichroism (ECD) and *Thermo*FAD measurements (Forneris et al. 2009; Pantoliano et al. 2001) were conducted as described previously (Graf et al. 2015). Differential scanning calorimetry (DSC) measurements were performed with 13.3 μM of each PDH isoform in 50 mM potassium phosphate buffer, pH 7.0. For data analysis and conversion, the Origin 7.0 SR4 software from OriginLab Corporation (Northampton, MA, USA) was used. Data points were fitted to non-two-state equilibrium-unfolding models by the Levenberg/Marquardt (LM) non-linear least square method. The obtained heat capacity (*C*_p_) is given in kcal mol^−1^ °C^−1^.

### Cofactor characterization

To determine whether FAD is covalently attached to the polypeptide chain, trichloroacetic acid (TCA)/acetone precipitation in combination with UV–Vis spectroscopy was used, which is described in detail elsewhere (Graf et al. 2015). To analyse the mass of the FAD cofactor, LC–ESI–MS was performed. Proteins were S-alkylated with iodoacetamide (5 mM for 30 min in the dark) and digested with modified trypsin in solution in 0.1 M ammonium bicarbonate buffer (Promega; Mannheim, Germany) overnight. Approx. 1 μg of each digest was loaded on a BioBasic C18 column (BioBasic-18, 150 × 0.18 mm, 5 μm; ThermoScientific, Waltham, MA, USA) using 0.1% formic acid (FA) as the aqueous solvent. A gradient of solvent B (5–32%) in solvent A was applied over 35 min, where solvent A is 0.1% FA in water and solvent B is 0.1% FA in acetonitrile. This was followed by a 15-min gradient from 32% B to 75% B to facilitate elution of large peptides, all at a flow rate of 1.5 μL min^−1^. Detection was performed with a QTOF MS (Bruker maxis 4G ETD) equipped with the standard ESI source in the positive ion, DDA mode, thereby switching to MSMS mode for eluting peaks. MS scans were recorded in a range of 150 to 2500 Da, and the six highest peaks were selected for fragmentation. Instrument calibration was performed using a commercial ESI calibration mixture (Agilent; Santa Clara, CA, USA). The analysis files were converted to mgf files using DataAnalysis (Bruker; Billerica, MA, USA), and a manual integration of the BPC of the desired masses was performed.

### Steady-state kinetic measurements

Apparent steady-state kinetic constants for all electron donors were measured using the standard ferrocenium assay. The oxidation of sugar substrates/electron donors was indirectly monitored by following the absorbance decrease of FC^+^ at 300 nm, pH 7.5, using an absorption coefficient of 4.3 mM^−1^ cm^−1^ (Kujawa et al. 2007). All measurements were performed in cuvettes of a reaction volume of 1 mL with the exception of maltotriose and allose, which were measured in cuvettes of 0.1 mL.

The steady-state oxygen reactivity was determined in 50 mM sodium phosphate buffer pH 7.4 at 30 °C using the fluorimetric Amplex Red/horseradish peroxidase assay (50 μM Amplex Red, 0.1 U mL^−1^ horseradish peroxidase) and 25 mM of the electron donor glucose in quadruplicates. Generated H_2_O_2_ (μM H_2_O_2_ min^−1^ mg^−1^ enzyme) was converted via a calibration curve into the oxygen reactivity of the enzyme (μM O_2_ min^−1^ mg^−1^ enzyme) (Krondorfer et al. 2014b).

Kinetic properties for the electron acceptors ABTS cation radical (ABTS^·+^), BQ and FC^+^ were measured with 25 mM glucose as electron donor at 30 °C. ABTS^·+^ was prepared by oxidizing 2,2′-azino-bis(3-ethylbenzothiazoline-6-sulphonic acid) with laccase (Galhaup et al. 2002; Hess et al. 2002) and subsequent removal of the enzyme by ultrafiltration. ABTS^·+^ was quantified by using its absorption coefficient. Electron acceptor concentrations were monitored in cuvettes with a reaction volume of 1 mL at the following wavelength, absorption coefficient and buffer: ABTS^·+^ at 414 nm, 36 mM^−1^ cm^−1^ (Childs and Bardsley 1975) and 100 mM sodium acetate buffer pH 4.0; BQ at 290 nm, 2.3 mM^−1^ cm^−1^ (Volc et al. 2001) and 100 mM sodium acetate buffer pH 4.0; and FC^+^ at 300 nm, 4.3 mM^−1^ cm^−1^ (Kujawa et al. 2007) and 100 mM sodium borate buffer pH 8.5.

### Analysis of GLC reaction products by GC-CI-QTOF MS

Detection of glucose and its reaction products after oxidation by the three *Am*PDH isoforms was conducted via gas chromatography in combination with chemical ionization quadrupole time-of-flight mass spectrometry (GC-CI-QTOF MS). A detailed description of the GC-CI-QTOF MS measurements can be found in Graf et al. (2015) and further details in Chu et al. (2015), Fiehn et al. (2000) and Vandendool and Kratz (1963).

## Results

### Expression and purification of the three *Am*PDH isoforms

**Fig. 1:**
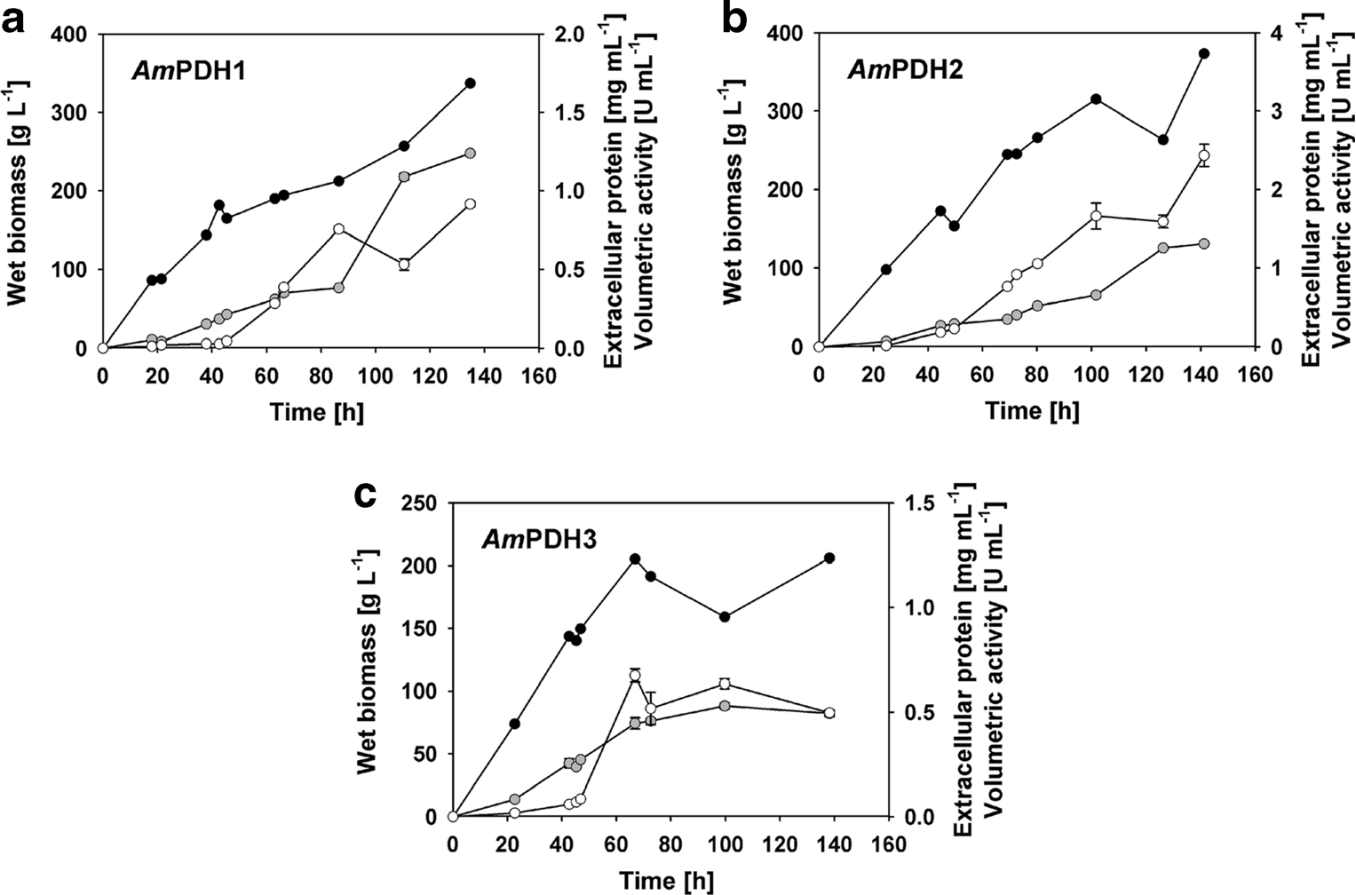
Time course of fermenter cultivations of recombinant *Pichia pastoris* for the production of **a** *Am*PDH1, **b** *Am*PDH2 and **c** *Am*PDH3. The cultivations were carried out in a 7-L aerated and stirred bioreactor with 4 L starting volume of basal salts cultivation medium. Wet biomass (*black circles*), extracellular protein (*grey circles*) and volumetric activity (*white circles*) are shown. Data are the mean of duplicate independent measurements ± the standard deviation indicated by the error bars

Heterologous production of the *Am*PDH isoforms in *P. pastoris* X33 in a 7-L stirred tank bioreactor as outlined in the “Materials and methods” section showed similar time courses with respect to biomass formation and extracellular protein concentration for *Am*PDH1 and *Am*PDH2 (Fig. 1). Extracellular activity was 2-fold higher for *Am*PDH2 throughout the cultivation as compared to *Am*PDH1. The levels of wet biomass, extracellular protein and PDH activity for the cultivation of *Am*PDH3 were comparable to that of *Am*PDH1 until 67 h of total cultivation time. From then on, however, the three parameters did not increase for the *Am*PDH3 cultivation as opposed to that of *Am*PDH1 and *Am*PDH2. This might have been caused by an excessive methanol feed, indicated by pO_2_ levels <20% at 70, 76 and 80 h of total cultivation time. Nevertheless, considerable *Am*PDH activity was obtained in the supernatant of all three cultivations (Supplementary Table S2), with total activities of 3500 U for *Am*PDH1, 8500 U for *Am*PDH2 and 1730 U for *Am*PDH3. Based on the specific activities of the purified enzymes (46.5 U mg^−1^ for *Am*PDH1, 37.5 U mg^−1^ for *Am*PDH2 and 10.0 U mg^−1^ for *Am*PDH3), between ~20 mg of recombinant protein per litre of medium (*Am*PDH1) and ~65 mg L^−1^ (*Am*PDH2) could be obtained in these cultivations.

**Fig. 2:**
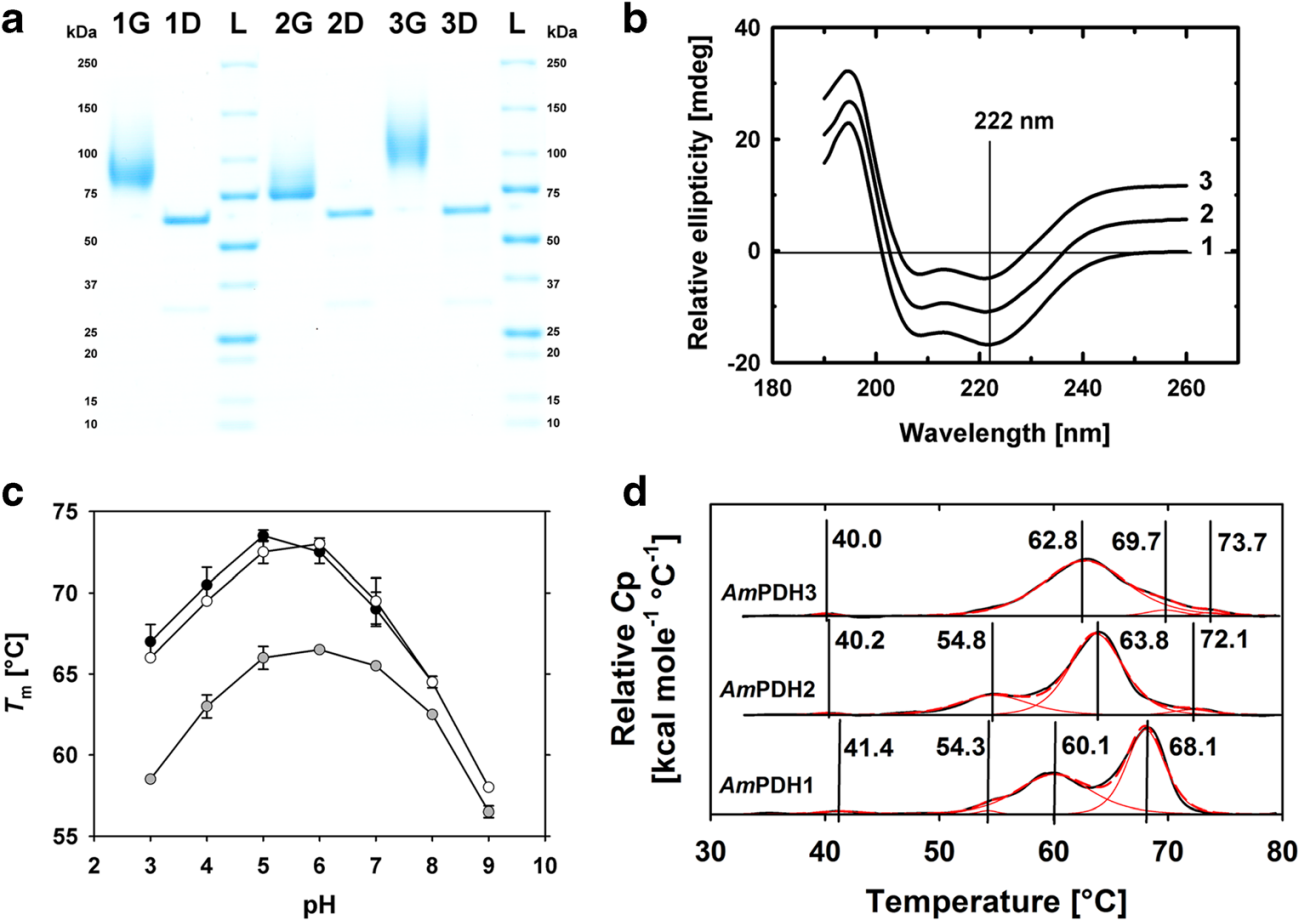
Biochemical and biophysical properties of *Am*PDH1, *Am*PDH2 and *Am*PDH3. **a** SDS-PAGE; *lanes* are numbered according to the corresponding *Am*PDH designation (*1* for *Am*PDH1, etc.), whereas *G* stands for glycosylated, *D* for deglycosylated and *L* for the molecular mass standard; the molecular mass of the standard’s individual bands is indicated at both sides of the figure in kilodalton. **b** Baseline-corrected electronic circular dichroism (ECD) spectra, normalized at 222 nm; labelling according to the *Am*PDH designation. To overlay the ECD spectra, the value 6 was added to all data points for *Am*PDH2 and 12 for *Am*PDH3, respectively. **c** *Thermo*FAD measurements of *Am*PDH1 (*black circles*), *Am*PDH2 (*grey circles*) and *Am*PDH3 (*white circles*). Data in **c** are the means of duplicate independent measurements ± the standard deviation indicated by the *error bars*. **d** Differential scanning calorimetry (DSC) curves obtained from experiments (*black line*) and individual peaks obtained from fitting (*red solid lines*) and overall fit (*red dashed line*). The maxima of the fitted peaks are indicated by *vertical lines*, and the corresponding melting temperature is indicated

The purification scheme described in the “Materials and methods” section and summarized in Supplementary Table S2 yielded the respective PDH isoforms with high apparent purity (Fig. 2a) and in amounts sufficient for subsequent characterization.

### Enzyme properties

As estimated by SDS-PAGE, the molecular mass of the three deglycosylated *Am*PDH isoforms is around 65 kDa (Fig. 2a, lanes with suffix ‘D’), which is in very good agreement with their theoretical molecular mass (Kittl et al. 2008). *Am*PDH3 showed the highest degree of glycosylation as judged by the broad, smeared band at 90–120 kDa on SDS-PAGE for the recombinant, glycosylated form (Fig. 2a, lane ‘3G’), which indicates heterogeneous N-glycosylation and also corresponds to the increased number of potential N-glycosylation sites of *Am*PDH3, as it has four or three additional sites when compared to *Am*PDH1 or *Am*PDH2, respectively (Kittl et al. 2008). As judged from far-UV ECD spectroscopy, the overall fold of the three proteins is similar (Fig. 2b) with distinct minima at 208 and 222 nm, indicating a predominant α-helical content (Barrow et al. 1992; Kelly et al. 2005). This is in good agreement with the secondary structure elements observed in the *Am*PDH1 crystal structure (Tan et al. 2013).

To assess the thermal stability of the three isoforms, their transition midpoint temperatures (*T*_m_) were recorded with two different methods. The increased intrinsic fluorescence of the FAD cofactor upon solvent exposure during protein denaturation was measured with the *Thermo*FAD assay (Forneris et al. 2009) over the pH range of 3 to 9 (Fig. 2c), while DSC was used to probe the *T*_m_ of the entire polypeptide and not only of a particular domain at pH 7 (Fig. 2d). Consequently, the *T*_m_ values derived from these two methods differed. The *Thermo*FAD-derived *T*_m_ values for *Am*PDH1 and *Am*PDH3 are very similar across the entire pH range (Fig. 2c, black and white circles). Both enzymes show their highest thermal stability between pH values 5 and 6, with *Am*PDH1 having its maximum *T*_m_ at 73.5 °C and pH 5 and *Am*PDH3 at 73.0 °C and pH 6. In contrast, *Am*PDH2 shows a significantly reduced thermostability over the whole pH range with a maximum *T*_m_ of 66.5 °C at pH 6 (Fig. 2c, grey circles).

For DSC measurements at pH 7, four peaks (two of these peaks were very minor) could be fitted to the measured thermograms for the three *Am*PDH isoforms (Fig. 2d). The peaks at *T*_m_ 68.1 °C (*Am*PDH1), 63.8 °C (*Am*PDH2) and 69.7 °C (*Am*PDH3) are in good agreement with the *Thermo*FAD-derived melting temperatures at pH 7 and most likely reflect the unfolding of the flavin domain. In addition, a second prominent denaturation peak was obtained at 60.1 °C for *Am*PDH1, 54.8 °C for *Am*PDH2 and 62.8 °C for *Am*PDH3, which could indicate the unfolding of another domain, possibly the substrate-binding domain. For *Am*PDH1, the measured values are in good agreement with recently published data (Krondorfer et al. 2014a), which reported a maximum *T*_m_ at 72 °C and pH 5–6 in *Thermo*FAD measurements as well as two main transitions at 57.8 °C and 65.3 °C at pH 7.5 during DSC measurements. Hence, the His_6_-tag does not influence the thermal stability of *Am*PDH1.

### FAD-related enzyme properties

**Fig. 3:**
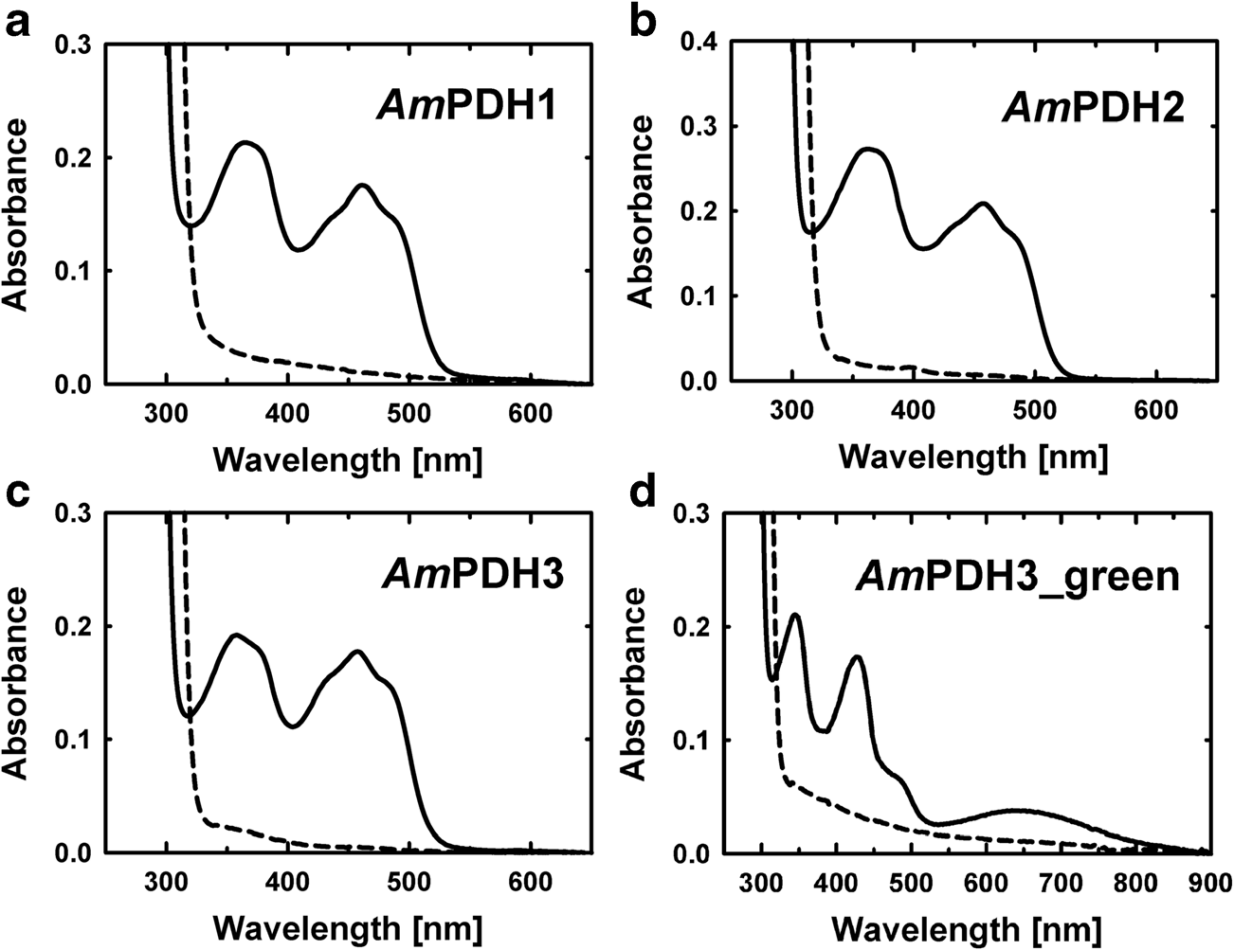
FAD-related enzyme properties of *Am*PDH1, *Am*PDH2 and *Am*PDH3. UV–Vis spectra of (i) the fully oxidized enzyme (*solid line*) and (ii) the corresponding trichloroacetic acid (TCA) precipitated enzyme solution (*dashed line*) of **a** *Am*PDH1, **b** *Am*PDH2, **c** *Am*PDH3 and **d** *Am*PDH3_green

Homology models with *Am*PDH1 as a template suggest that the FAD cofactor is also covalently tethered via His-102 both in *Am*PDH2 and in *Am*PDH3 (Supplementary Fig. S2), which was further corroborated by protein precipitation using 10% TCA and 40% acetone. *Am*PDH1 was also subjected to the same treatment and served as positive control, as it was shown to have a covalently attached FAD (Krondorfer et al. 2014a; Tan et al. 2013). UV–Vis spectra of the fully oxidized proteins were recorded before and after precipitation (Fig. 3a–c). Before precipitation, the UV–Vis spectra of the three isoforms are characteristic for a flavin-dependent enzyme with absorption maxima at 365 and 461 nm for *Am*PDH1, 362 and 458 nm for *Am*PDH2 and 358 and 458 nm for *Am*PDH3. An absorption peak at 450 nm is characteristic of free FAD in solution (Macheroux 1999), which is red shifted for all three isoforms. This bathochromic shift is caused by more extensive delocalization of the π-electron system of the aromatic isoalloxazine ring system and suggests covalent attachment to the protein (Schleicher et al. 2007). After TCA/acetone precipitation, <3% FAD was found in the supernatant of all three isoforms, which unequivocally proves the presence of a covalently tethered FAD cofactor, which is in agreement with the homology models for *Am*PDH2 and *Am*PDH3 (Supplementary Fig. S2).

Further characterization of the three isoforms revealed that the absorption around 460 nm completely disappeared upon reduction with glucose (Supplementary Fig. S3a–c), which is a characteristic for flavin-dependent enzymes. Interestingly, a second form of *Am*PDH3 was identified, which was subsequently termed *Am*PDH3_green based on its green colour. *Am*PDH3_green was obtained by batch cultivations of the same *P. pastoris* expression host that was also used for production of the standard ‘yellow’ form of *Am*PDH3, albeit the cultivation was performed with a complex medium containing yeast extract and peptone (rather than the basal salt medium used in the other *Pichia* fermenter cultivations) in Duran GLS 80 table-top fermenters (Duran Group; Wertheim/Main, Germany). The UV–Vis spectrum of fully oxidized *Am*PDH3_green shows two distinct peaks at 342 and 427 nm, a broad peak with a maximum at around 640 nm and a shoulder at approximately 486 nm (Fig. 3d, Supplementary Fig. S3d). Moreover, TCA/acetone precipitation revealed that 88% of FAD is covalently attached to *Am*PDH3_green (Fig. 3d). The flavin domain of a related GMC member, cellobiose dehydrogenase (CDH), carries a non-covalently attached FAD. Igarashi and coworkers showed that the flavin domain of CDH from *Humicola insolens* (*Hi*CDH) also has a green colour and that it predominantly (~60%) contains 6-hydroxy-FAD (Igarashi et al. 1999). When superimposing the oxidized UV–Vis spectra of *Am*PDH3_green and *Am*PDH3 with those of the flavin domains of *Hi*CDH and CDH from *Phanerochaete chrysosporium* (*Pc*CDH), the latter containing only FAD, the spectra of *Am*PDH3_green and *Hi*CDH as well as *Am*PDH3 and *Pc*CDH matched very well (Supplementary Fig. S3e, f). The spectra of both *Am*PDH3 and *Pc*CDH show the spectral characteristics routinely observed for flavoenzymes (Macheroux 1999), whereas the UV–Vis spectra of *Hi*CDH and *Am*PDH3_green are typical for 6-hydroxyflavin (Igarashi et al. 1999). Furthermore, the presence of 6-hydroxy-FAD with a mass of +16 Da, which is indicative for an additional oxygen atom, was found by mass spectrometry. We measured the FAD-linked peptide (VLGGCSS**H**NSMVYTR + FAD, mass 1667.7628 + 783.16 = 2450.922 Da) and the oxidized FAD-linked peptide (VLGGCSS**H**NSMVYTR + FAD + oxygen, mass 1667.7628 + 783.16 + 16 = 1466.922 Da) and found less than 2% of the oxidized FAD-linked peptide in the standard *Am*PDH3 preparation, while this fraction was approx. 40% for the *Am*PDH3_green preparation. Based on these MS data, we estimate that the flavin cofactor in the enzyme preparation termed *Am*PDH3_green and obtained by batch cultivations of *P. pastoris* on the complex medium is a mixture of ~40% *Am*PDH3 containing 6-hydroxy-FAD and ~60% *Am*PDH3 containing FAD. It is thought that 6-hydroxy-FAD is formed from FAD as a result of a redox reaction in the presence of oxygen (Marshall et al. 2005), and we propose that use of the complex medium for the aerobic production of *Am*PDH3 favoured this reaction.

### pH and temperature dependence of activity

**Fig. 4:**
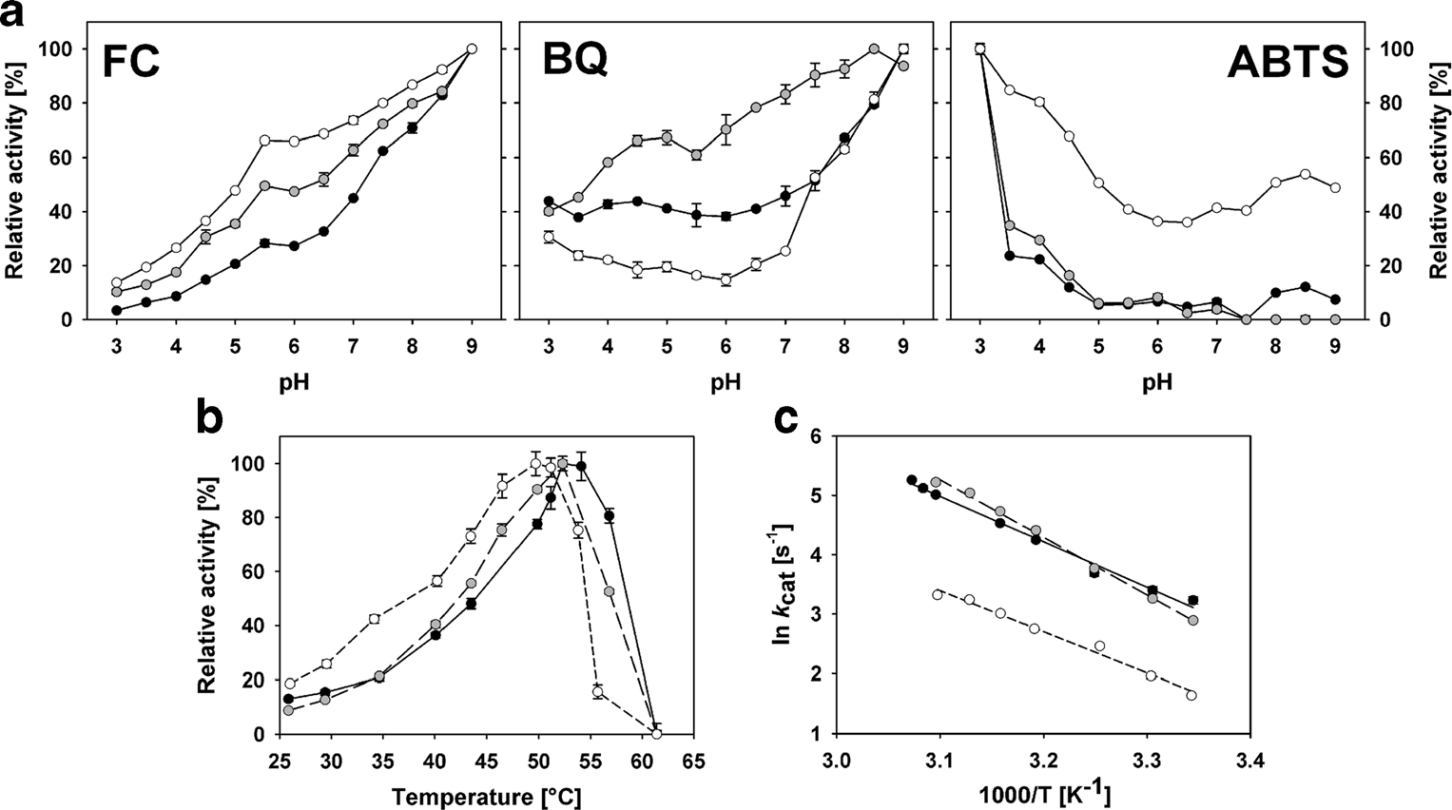
Influence of the pH and the temperature on the activities of *Am*PDH1 (*black circles*), *Am*PDH2 (*grey circles*) and *Am*PDH3 (*white circles*). **a** Relative activity at different pH values for *FC* ^*+*^, *BQ* and *ABTS* ^*·+*^ as electron acceptor and as indicated in the upper corners of the plots. **b** Relative activity at different temperatures and **c** the corresponding Arrhenius plots for *Am*PDH1 (*solid line*), *Am*PDH2 (*long-dashed line*) and *Am*PDH3 (*short-dashed line*). The activation energies of the transition states (*E*_a_) were 63.9 kJ mol^−1^ for *Am*PDH1, 80.8 kJ mol^−1^ for *Am*PDH2 and 57.4 kJ mol^−1^ for *Am*PDH3. Data are the mean of duplicate (**a**) or triplicate (**b**, **c**) independent measurements ± the standard deviation indicated by the *error bars*

The pH dependence of *Am*PDH activity was tested for the electron acceptor substrates FC^+^, BQ and ABTS^·+^ in the pH range of 3–9 with glucose as electron donor (Fig. 4a). For FC^+^, the pH optimum was in the alkaline region at pH 9 for all three isoforms. Here, it should be noted that the standard ferrocenium assay for monitoring *Am*PDH activity was conducted at pH 7.5, at which *Am*PDH1 shows ~62%, *Am*PDH2 ~72% and *Am*PDH3 ~80% of their maximum relative activity. The pH optimum for BQ was at pH 9 for *Am*PDH1 and *Am*PDH3 and at pH 8.5 for *Am*PDH2. Both *Am*PDH1 and *Am*PDH2 showed a very broad pH/activity curve with significant activity (>40% relative activity) over the entire pH range measured. The relative activity with ABTS^·+^ was highest at pH 3 for all isoforms. *Am*PDH3 showed pronounced relative activity (>40%) over the entire pH range considered with this substrate.

The temperature dependence of the activity was determined with the standard ferrocenium assay for 3 min between 25 and 62 °C (Fig. 4b). The highest activities were observed at 52 °C for *Am*PDH1 and *Am*PDH2 and at 50 °C for *Am*PDH3. After reaching the maximum activity, it rapidly decreased with increasing temperature and was completely abolished at 61 °C for the three isoforms. These values for the maximum activity are significantly lower than *T*_m_ values measured by DSC and indicate that activity is lost well before complete unfolding of a domain or the entire polypeptide; hence, turnover stability is lower than thermal stability. The slope of the corresponding Arrhenius plots (Fig. 4c) was used to calculate the transition states activation energies (*E*_a_), which were 63.9 kJ mol^−1^ for *Am*PDH1, 80.8 kJ mol^−1^ for *Am*PDH2 and 57.4 kJ mol^−1^ for *Am*PDH3. The *E*_a_ values clearly demonstrate that the temperature dependency of the reaction rate constants is highest for *Am*PDH2 and lowest for *Am*PDH3.

### Kinetic properties for electron donors and GLC reaction products

Apparent steady-state kinetic constants were determined for *Am*PDH1, *Am*PDH2 and *Am*PDH3 for the following sugar substrates (electron donors) by varying their concentration and keeping the concentration of the electron acceptor FC^+^ constant at 0.2 mM (Table 1): d-glucose (GLC), maltose (MAL), maltotriose (MTR), methyl-α-d-glucopyranoside (MGP), d-mannose (MAN), d-allose (ALL), d-galactose (GAL), d-xylose (XYL) and lactose (LAC). Recombinant His_6_-tagged *Am*PDH1, which was used for this study, gave essentially identical kinetic constants as *Am*PDH1 isolated from its natural fungal source (Sygmund et al. 2008) and untagged recombinant enzyme expressed in *P. pastoris* (Sygmund et al. 2012) for several substrates including GLC, GAL, XYL and LAC, indicating that the His_6_-tag and the altered glycosylation do not affect the reactivity of PDH.

**Table 1:** Apparent steady-state kinetic constants for several electron donor substrates with the ferrocenium cation as electron acceptor

Electron donor	*Am*PDH1			*Am*PDH2			*Am*PDH3		
	*k*_cat_ [s^−1^]	*K*_m_ [mM]	*k*_cat_/*K*_m_ [mM^−1^ s^−1^]	*k*_cat_ [s^−1^]	*K*_m_ [mM]	*k*_cat_/*K*_m_ [mM^−1^ s^−1^]	*k*_cat_ [s^−1^]	*K*_m_ [mM]	*k*_cat_/*K*_m_ [mM^−1^ s^−1^]
GLC	42.2 ± 0.2	0.82 ± 0.01	51.5	35.7 ± 0.2	0.53 ± 0.01	67.4	8.16 ± 0.11	3.28 ± 0.18	2.48
MAL	44.7 ± 0.4	10.3 ± 0.3	4.34	7.63 ± 0.08	11.6 ± 0.5	0.658	0.48 ± 0.01	2.22 ± 0.11	0.216
MTR	35.9 ± 2.2	62.5 ± 9.0	0.574	21.2 ± 0.7	54.6 ± 6.3	0.388	16.3 ± 1.0	154 ± 19	0.106
MGP	41.5 ± 0.8	0.66 ± 0.05	62.9	85.0 ± 0.6	2.00 ± 0.06	42.5	1.00 ± 0.01	0.95 ± 0.06	1.05
MAN	26.5 ± 1.1	94.0 ± 9.6	0.282	109 ± 1.4	34.9 ± 2.0	3.12	34.7 ± 0.6	185 ± 11	0.188
ALL	18.4 ± 0.5	18.3 ± 1.8	1.01	40.2 ± 1.9	113 ± 13	0.356	35.3 ± 1.0	181 ± 11	0.195
GAL	34.3 ± 3.0	0.64 ± 0.13	53.6	137 ± 1.9	40.1 ± 1.6	3.41	19.3 ± 0.4	29.7 ± 2.3	0.650
XYL	51.3 ± 1.6	2.39 ± 0.14	21.5	146 ± 1.8	17.0 ± 0.8	8.59	29.5 ± 0.3	9.34 ± 0.33	3.16
LAC	33.7 ± 6.1	76.7 ± 7.9	0.439	209 ± 13	2288 ± 168	0.0913	75.3 ± 2.0	235 ± 14	0.320

The measurements were done with the ferrocenium cation at a fixed concentration of 0.2 mM in 50 mmol Na phosphate buffer pH 7.5 and at 30 °C. Data are the mean of triplicate independent measurements ± the standard deviation

*GLC* d-glucose, *MAL* maltose, *MTR* maltotriose, *MGP* methyl-α-d-glucopyranoside, *MAN* d-mannose, *ALL* d-allose, *GAL* d-galactose, *XYL* d-xylose, *LAC* lactose

As judged from the catalytic efficiencies *k*_cat_/*K*_m_, GLC is among the preferred sugar substrates for all three isoforms. Previous experimental and computational studies demonstrated that *Am*PDH1 can oxidize GLC at C2 and C3, and when extending the reaction time, double oxidation of this sugar at both C2 and C3 occurs (Graf et al. 2013, 2015; Sedmera et al. 2006), with a clear preference for the 2-keto-GLC intermediate. Gas chromatography in combination with chemical ionization time-of-flight mass spectrometry (GC-CI-TOF MS) showed that *Am*PDH2 and *Am*PDH3 double oxidize GLC to 2,3-diketo-GLC as well and that this reaction proceeds mainly via the 2-keto-GLC intermediate (Supplementary Table S3).

*Am*PDH1 has an open active site geometry, and interaction with its carbohydrate substrates occurs mainly at the subsite closest to the FAD where oxidation takes place (referred to as subsite C, catalytic site). The adjacent subsite, named B1 (‘B’ for binding), accommodates additional sugar moieties of oligosaccharide substrates, yet oligomeric substrates form only few binding interactions in subsite B1 (Tan et al. 2013). This is also reflected by the catalytic efficiencies *k*_cat_/*K*_m_ or the substrate selectivity, i.e. the ratio of the catalytic efficiencies for two substrates, calculated for the substrate series glucose–maltose–maltotriose. The substrate selectivity of glucose over maltose is 11.9 [(*k*_cat_/*K*_m_)_GLC_/*k*_cat_/*K*_m_)_MAL_] or 89.7 for maltotriose, indicating strong preference for the monomeric over oligomeric sugar substrates. Substrate selectivities of glucose over maltose/maltotriose are comparable for *Am*PDH3 (11.3 and 22.7 for maltose and maltotriose, respectively), while *Am*PDH2 discriminates more strongly between these sugars, with selectivity values of 102 and 173 for MAL and MTR, respectively.

A possible explanation for this behaviour could be the replacement of Val-511 in *Am*PDH1 by a slightly bulkier leucine (Leu-509) in *Am*PDH2. This leucine could interfere more pronouncedly with binding of di- or oligosaccharides than valine, as was shown for steric clashes of Val-511 when maltose was positioned in the active site of *Am*PDH1 for certain oxidation modes (Tan et al. 2013). This position in the active site is taken up by the even bulkier amino acid tryptophan, Trp-509, in *Am*PDH3 (Supplementary Fig. S2), and one would anticipate an even stronger interference with sugar binding. However, in a previous study employing MD simulations, we could demonstrate that in the *Am*PDH1 variant V511W Trp-511 is bent out of the active site by forming H bonds to the backbone oxygen atom of Asp-90 and Pro-92 and, hence, does not interfere with sugar binding (Graf et al. 2015). For *Am*PDH3, a similar mechanism might be responsible to more easily accommodate larger substrates such as MAL.

Next, we tested the reactivity of the three *Am*PDH isoforms with the C2, C3 and C4 epimers of glucose, i.e. MAN, ALL and GAL, as well as with XYL, lacking the exocyclic CH_2_OH group at C5. Previous characterization of PDH from another source, *Agaricus xanthoderma* (*Ax*PDH), showed that orientation of the hydroxyl group at C2 and C3 is important for substrate specificity and reactivity, whereas the position of the OH group (axial or equatorial) at C4 as in galactose or the presence of the exocyclic CH_2_OH group is of lesser importance (Kujawa et al. 2007). While these observations are, in general, also valid for the *Am*PDH isoforms, they also show some interesting differences with respect to substrate selectivity. As judged from the catalytic efficiencies, GLC, GAL and XYL are good substrates of *Am*PDH1, while MAN and ALL are poor ones. In contrast to *Ax*PDH, which does not show any reactivity with these two latter sugars, *Am*PDH1 shows low reactivity with both sugars. Both *Am*PDH2 and *Am*PDH3 give catalytic efficiencies for MAN that are lower by one order of magnitude than the corresponding value for GLC, yet both enzymes show *k*_cat,MAN_ values that are significantly higher than the *k*_cat,GLC_ value, indicating that MAN is a good substrate when present in saturating conditions. Catalytic efficiencies for ALL are low for all three isoforms, demonstrating again the importance of the orientation of the OH group at C3. Overall, the three *Am*PDH isoforms show broad sugar substrate promiscuity and react with the same sugar substrates with distinct differences when it comes to relative activities. All three *Am*PDH isoforms oxidize the monosaccharides GLC, MAN, GAL and XYL, which are important constituents of cellulose and hemicelluloses, with varying efficiencies but with appreciable reactivity. This indicates that these lignocellulose-derived sugars could be in vivo substrates of the *Am*PDH isoforms during growth of the fungus on plant material.

We also determined the apparent kinetic constants for the preparation of *Am*PDH3_green. While the apparent Michaelis constants were comparable to *Am*PDH3 for all tested sugars, the *k*_cat_ values for these sugars were determined to be in the range of approximately 25 to 35% of the corresponding values measured for the standard ‘yellow’ preparation of *Am*PDH3. We estimated that the preparation of *Am*PDH3_green contains ~40% 6-hydroxy-FAD and ~60% FAD. Hence, the activity measured in this preparation can be mainly attributed to the standard form of *Am*PDH3 containing FAD, while the catalytic activity of the modified form carrying 6-hydroxy-FAD seems to be very low to negligible. This is in accordance with reports on other flavin-dependent enzymes that were found to contain 6-hydroxy-FAD (Igarashi et al. 1999; Tedeschi et al. 1994; Zanetti et al. 1983). For example, ferredoxin-NADP^+^ reductase reconstituted with 6-hydroxy-FAD showed 4–14% relative activity compared to the enzyme containing FAD (Zanetti et al. 1983), while the flavin domain of *Hi*CDH reconstituted with 6-hydroxy-FAD resulted in 16–35% relative activity (Igarashi et al. 1999).

### Kinetic properties for electron acceptors

Cultivation of *A. meleagris* under conditions of oxygen depletion resulted in different levels of transcription for the three isoforms (Kittl et al. 2008), which could indicate different reactivities towards oxygen. Consequently, the steady-state oxygen reactivity of the three isoforms was determined. The oxygen reactivity had recently been reported for *Am*PDH1 as 0.095 ± 0.003 μmol min^−1^ mg^−1^ and, therefore, practically negligible (Krondorfer et al. 2014b). The recombinant His_6_-tagged *Am*PDH1, which was used in the current study, showed the identical value for O_2_ reactivity as the untagged recombinant enzyme, indicating that the hexa-histidine tag does not affect O_2_ reactivity. Oxygen reactivities of the other two isoforms were 0.115 ± 0.006 μmol min^−1^ mg^−1^ for *Am*PDH2, 0.105 ± 0.002 μmol min^−1^ mg^−1^for *Am*PDH3 and 0.100 ± 0.003 μmol min^−1^ mg^−1^for *Am*PDH3_green. Thus, the three isoforms show very low activity with oxygen, indicating that it is a physiologically irrelevant electron acceptor and that all assays can be conducted at aerobic conditions.

The exact biological function of PDH is not known to date, but a role in detoxification of quinones or in preventing repolymerization of radical intermediates formed during ligninolysis was suggested (Peterbauer and Volc 2010). Recently, an additional function for GMC oxidoreductases including PDH was proposed, namely the reduction of quinones derived from lignocellulose or secreted by fungal organisms or complexed metal ions (Kracher et al. 2016). These reduced diphenols/quinones/metal ions, in turn, can act as redox mediators and shuttle electrons to lytic polysaccharide monooxygenases (LPMO), copper-dependent enzymes that are involved in the degradation of recalcitrant polysaccharides including cellulose and hemicellulose (Beeson et al. 2015; Horn et al. 2012). By continuously supplying the reduced form of low-molecular mass electron donors that are needed by LPMO, GMC oxidoreductases including PDH could thus play an indirect role in polysaccharide degradation. Therefore, we determined the apparent steady-state kinetic constants of the *Am*PDH isoforms for three chemically distinct model electron donor/acceptor substrates, the ABTS cation radical (ABTS^·+^, a 1-e^−^ acceptor), 1,4-benzoquinone (BQ, 2-e^−^) and the ferrocenium cation (FC^+^, 1-e^−^), with 25 mM GLC as electron donor (Table 2). Recombinant His_6_-tagged *Am*PDH1, which was used for this study, had essentially the same kinetic constants for BQ and FC^+^ as the untagged recombinant enzyme reported by Sygmund et al. (2012), again indicating that the tag does not influence its reactivity towards these electron acceptors.

**Table 2:** Apparent steady-state kinetic constants for electron acceptors with d-glucose as electron donor

Electron acceptor	pH	*Am*PDH1			*Am*PDH2			*Am*PDH3		
		*k*_cat_ [s^−1^]	*K*_m_ [mM]	*k*_cat_/*K*_m_ [mM^−1^ s^−1^]	*k*_cat_ [s^−1^]	*K*_m_ [mM]	*k*_cat_/*K*_m_ [mM^−1^ s^−1^]	*k*_cat_ [s^−1^]	*K*_m_ [mM]	*k*_cat_/*K*_m_ [mM^−1^ s^−1^]
ABTS^·+^	4.0	7.57 ± 0.94	0.0791 ± 0.009	94.6	4.28 ± 0.25	0.109 ± 0.010	38.9	5.32 ± 0.12	0.0132 ± 0.0012	532
BQ	4.0	65.4 ± 5.5	1.38 ± 0.28	47.4	23.4 ± 0.5	0.0325 ± 0.0019	780	3.57 ± 0.11	1.88 ± 0.14	1.89
FC^+^	8.5	130 ± 11	0.161 ± 0.038	812	48.1 ± 1.1	0.0201 ± 0.0023	2400	9.86 ± 0.19	0.0103 ± 0.0009	986

Measurements were done with d-glucose as electron donor at a fixed concentration of 25 mM and at 30 °C. Data are the mean of triplicate independent measurements ± the standard deviation. Measurements were performed in 100 mM Na acetate buffer pH 4.0 for ABTS^·+^ and BQ and in 100 mM Na borate buffer pH 8.5 for FC^+^

*ABTS* ^*·+*^ (2,2′-azino-bis(3-ethylbenzothiazoline-6-sulphonic acid) cation radical), *BQ* (1,4-benzoquinone), *FC* ^*+*^ (ferrocenium cation)

The *Am*PDH isoforms showed distinct differences in their kinetic properties with these different electron acceptor substrates. The catalytic efficiency of *Am*PDH3 for ABTS^·+^ was significantly higher than that of the other two isoforms, which can mainly be attributed to its low *K*_M_ value. When using BQ as substrate, *Am*PDH2 showed the highest catalytic efficiency among the three isoforms, which was approximately one or two orders of magnitude higher when compared to *Am*PDH1 and *Am*PDH3, respectively. The clear preference of *Am*PDH2 for BQ was again mainly caused by *K*_M,BQ_. For FC^+^, the catalytic efficiency of *Am*PDH2 was about 3.5-fold higher compared to the other two isoforms. *Am*PDH1 and *Am*PDH3 had similar catalytic efficiencies, with the latter showing lower *K*_M,FC_ and *k*_cat,FC_ values. The *K*_M_ values for *Am*PDH3_green were comparable to *Am*PDH3 for all tested electron acceptors, whereas the corresponding *k*_cat_ values were significantly lower (20 to 25%) than those of *Am*PDH3. These kinetic properties determined for the *Am*PDH isoforms with model substrates—a radical, a quinone and a complexed iron ion—indicate that the individual enzymes have different preferred electron acceptor substrates.

## Discussion

Kittl et al. (2008) isolated three genes from *A. meleagris* that were putatively assigned as pyranose dehydrogenase genes *pdh1*, *pdh2* and *pdh3*. Whereas the enzyme *Am*PDH1 had been thoroughly studied, no biochemical or biophysical data were available on the other two putative isoforms, *Am*PDH2 and *Am*PDH3. Both genes, *pdh2* and *pdh3*, could be successfully cloned into the expression vector pPICZB and heterologously expressed in *P. pastoris*. Characterization of *Am*PDH2 and *Am*PDH3 and comparison with *Am*PDH1 showed that these three isoforms show similar secondary structure elements, carry a covalently tethered FAD cofactor and show UV–Vis spectra characteristic for flavin-dependent proteins resulting in their yellow colour. For *Am*PDH3, a green-coloured form was obtained as well, which showed a UV–Vis spectrum indicative for 6-hydroxy-FAD. This is supported by MS data, which confirmed the presence of an FAD with an increased mass of 16 Da, indicating an oxygen atom. We propose that 6-OH-FAD is formed in the presence of oxygen and only when using a complex medium for the aerobic production of *Am*PDH3.

The three isoforms show comparable substrate specificity and kinetic properties with respect to their electron donor sugar substrates. They oxidize a range of different monosaccharides and oligosaccharides including GLC, MAN, GAL and XYL, which are important building blocks of cellulose and hemicelluloses. All three isoforms oxidize GLC both at C2 and C3, and upon prolonged reaction, C2 and C3 double-oxidized glucose is the product, confirming that the *A. meleagris* genes *pdh2* (AY753308.1) and *pdh3* (DQ117577.1) indeed encode pyranose dehydrogenases. While reactivity with electron donor substrates was comparable for the three AmPDH isoforms, their kinetic properties differed significantly for the model electron acceptor substrates tested, a radical (the ABTS cation radical), a quinone (benzoquinone) and a complexed iron ion (ferrocenium ion).

Thus, a possible explanation for this multiplicity could be that in vivo the different PDH isoforms react preferentially with structurally different electron acceptors. *A. meleagris* is a litter-decomposing basidiomycete, the genome sequence of which has not been deduced to date. In contrast, the genome of the closely related organism *A. bisporus* was published recently (Morin et al. 2012). *A. bisporus* is a model organism for adaptation, persistence and growth in a humic-rich environment. It is found over a wide geographical range, growing on leaf and needle litter in various temperate forests or even pastoral land use areas. It shares this humicolous ecological niche with at least 200 other species of *Agaricus* (Kerrigan et al. 2013) including *A. meleagris*. *A. bisporus* has a large set of genes encoding carbohydrate-active enzymes acting on plant cell wall polysaccharides, including a complete set of enzymes degrading crystalline cellulose and xylans. Furthermore, it has a number of lignin-converting oxidoreductase genes including a limited set of ligninolytic peroxidases compared to wood-degrading basidiomycetes. In contrast, the *A. bisporus* genome contains a large set of heme-thiolate peroxidase genes, including aromatic peroxygenases (APOs) and classic chloroperoxidases (CPOs), showing a significant expansion with these genes relative to wood decay fungi. The expansion of heme-thiolate peroxidases together with that of β-etherases was suggested to be responsible for a broad attack on decaying lignin and related metabolites found in a humic acid-rich environment by *A. bisporus*. Interestingly, pyranose dehydrogenase was also found among the protein families in expansion in *A. bisporus*. Gene expression of the *pdh* gene in *A. bisporus* was significantly upregulated in the presence of humic substances (Morin et al. 2012).

Humic substances are a mixture of substituted aromatic rings, heterocycles and aliphatic side chains, which are cross-linked by oxygen and nitrogen groups, with hydroxyl, carboxyl, amino, phenolic and quinone groups as the major functionalities. In addition, humic material has a relatively high number of free radicals, and it is known to contain complexed iron species (Van Trump et al. 2006). Humic substances can be used as an effective electron sink by various microorganisms, transferring electrons to quinone moieties, metal ions or other redox-active functional groups present (Piepenbrock and Kappler 2013). Furthermore, they have been shown to act as natural electron shuttles or redox mediators (Van Trump et al. 2006). Currently, the natural electron acceptor of PDH is not known. Since PDH is an extracellular enzyme secreted by litter-decomposing fungi that live in humic-rich environment, it is conceivable that humic substances act as an electron acceptor. In fact, humic substances could play a role as redox mediators between PDH and lytic polysaccharide monooxygenases, which are frequently found in litter-degrading or coprophilous fungi such as *A. bisporus* (11 putative *lpmo* genes) or *C. cinerea* (30 putative *lpmo* genes). Interestingly, *C. cinerea* contains six putative GMC *pdh* genes (Kracher et al. 2016; Stajich et al. 2010), which could be involved in these electron transfer reactions in a similar fashion.

## Electronic supplementary material

ESM 1 (PDF 3962 kb)
