# Supplementary material for: Characterization of three pyranose dehydrogenase isoforms from the litter-decomposing basidiomycete Leucoagaricus meleagris (syn. Agaricus meleagris)
Source: Appl Microbiol Biotechnol. 2016 Dec 19;101(7):2879–91. doi: 10.1007/s00253-016-8051-1 (PMC5352738; doi:10.1007/s00253-016-8051-1)
Supplement: Supplementary file 1 — (PDF 3962 kb) [file 253_2016_8051_MOESM1_ESM.pdf]

Journal: Applied Microbiology and Biotechnology

Characterisation of three pyranose dehydrogenase isoforms from the litter-decomposing basidiomycete *Leucoagaricus meleagris* (syn. *Agaricus meleagris*)

Michael M.H. Graf[1,2], Sandra Weber[1], Daniel Kracher[1,2], Roman Kittl[1], Christoph Sygmund[1], Roland Ludwig[1,2], Clemens Peterbauer[1,2], Dietmar Haltrich[1,2]

[1]Food Biotechnology Laboratory, Department of Food Science and Technology, University of Natural Resources and Life Sciences (BOKU), Muthgasse 18, 1190 Vienna, Austria
[2]BioToP – The Doctoral Programme on Biomolecular Technology of Proteins, Muthgasse 18, 1190 Vienna, Austria

Address correspondence to:
Prof. Dietmar Haltrich
Department of Food Science and Technology
University of Natural Resources and Life Sciences (BOKU)
Muthgasse 18, 1190 Vienna, Austria.
Tel.: +43-1-47654-75211; **Fax:** +43-1-47654-75039; **E-mail**: dietmar.haltrich@boku.ac.at

Table S1 Primer sequences for removing the *c-myc* epitope of the pPICZB vector and for adding the *Not*I- or *BstB*I- and the *Xba*I restriction sites to the 3'- and 5'-ends of the *pdh1, pdh2,* and *pdh3* gene, respectively. Restriction sites are highlighted in bold, underlined letters.

Name	Sequence (5'–3')
pPICZB-6His-fw	CATCATCATCATCATCATTGAGTTTGTAGCCTTAGACATG
pPICZB-6His-*Xba*I-rv	ATGATGATGATGATGATG**TCTAGA**AAGCTGGCGGCCGCCGCGGC
*Am*PDH1-*Not*I-fw	AAA**GCGGCCGC**ATGCTGCCTCGAGTGACCAAGTTG
*Am*PDH1-*Xba*I-rv	TTT**TCTAGA**GTTATAACTCTTTGCTATCAACGC
*Am*PDH2-*BstB*I-fw	AAA**TTCGAA**ACGATGCTCTCTCGAGTGGCCAAG
*Am*PDH2-*Xba*I-rv	TTT**TCTAGA**GTTGTAGCTATCCGCGATCAA
*Am*PDH3-*BstB*I-fw	AAA**TTCGAA**ACGATGCTCCCTCGAGTGGCCAGG
*Am*PDH3-*Xba*I-rv	TTT**TCTAGA**GGCATAGCTCTTTGCTATCAA

Table S2 Purification of recombinantly produced *Am*PDH1, *Am*PDH2, and *Am*PDH3. Abbreviations: SUP, crude culture supernatant; HIC, hydrophobic interaction chromatography; IMAC, immobilized-metal affinity chromatography; $IMAC_{P1}$ depicts pool 1 with highest purity, which was used for subsequent characterizations, and $IMAC_{P2}$ pool 2 with lower purity; IEX, ion exchange chromatography.

Enzyme	Purification step	Volume [mL]	Protein [mg]	Activity [U]	Specific activity [U/mg]	Recovery [%]	Purification [-fold]
*Am*PDH1	SUP	3,800	4,700	3,500	0.74	100	1.00
	HIC	440	-	2,800	-	80.0	-
	IMAC	1.90	58.1	2,700	46.5	77.1	62.8
*Am*PDH2	SUP	3,500	4,590	8,500	1.85	100	1.00
	HIC	600	-	7,800	-	91.8	-
	$IMAC_{P1}$	1.90	108	4,050	37.5	47.6	20.3
	$IMAC_{P2}$	1.90	48.9	1,750	35.8	20.6	19.4
*Am*PDH3	SUP	3,500	1,730	1,740	1.01	100[a]	1.00
	HIC	640	-	3,630	-	208	-
	IEX	200	-	2,850	-	164	-
	IMAC	1.90	220	2,200	10.0	126	9.90

[a] *Am*PDH3 was inhibited by an unknown component in the culture supernatant, after removal of this component the activity increased

Table S3 Analysis of reaction products of glucose oxidation by *Am*PDH isoforms analysed gas chromatography in combination with chemical ionization time-of-flight mass spectrometry (GC-CI-TOF MS), the methods is described in in detail Graf et al. (2015)

Enzyme	Reaction time (min)	Concentration (mM)		Relative (mM)	concentration	Relative ratio (%)			
		glucose	2-keto glucose	3-keto glucose	2.3-diketo glucose	glucose	2-keto glucose	3-keto glucose	2.3-diketo glucose
*Am*PDH1	3	2.689	1.720	0.000	0.061	60.2	38.5	0.0	1.4
	8	2.432	1.686	0.001	0.273	55.4	38.4	0.0	6.2
	120	0.427	1.331	0.026	1.271	14.0	43.6	0.9	41.6
*Am*PDH2	3	2.728	0.087	0.001	0.012	96.5	3.1	0.0	0.4
	8	2.543	0.346	0.000	0.093	85.3	11.6	0.0	3.1
	120	0.065	0.117	0.015	0.689	7.3	13.2	1.7	77.8
*Am*PDH3	3	2.773	0.086	n.d	n.d	97.0	3.0	n.d.	n.d.
	8	2.500	0.165	n.d	0.010	93.5	6.2	n.d.	0.4
	120	1.963	0.921	0.022	0.357	60.2	28.2	0.7	10.9
*Am*PDH3 _green	3	3.299	0.073	0.001	0.000	97.8	2.2	0.0	0.0
	8	3.318	0.245	0.000	0.006	93.0	6.9	0.0	0.2
	120	2.744	1.727	0.001	0.371	56.7	35.7	0.0	7.7

2-Ketoglucose and glucose were analyzed and quantitated via external calibration. Concentrations of these compounds given in the table were in 1 mL aliquots samples. For 3-ketoglucose and 2,3-diketoglucose, calculation of semiquantitative results was based on the ratio of peak area of the appropriate analyte to 2-ketoglucose at a given mass-to-charge ratio.

Fig. S1 Multiple sequence alignment of the amino acid sequences from *Am*PDH1, *Am*PDH2, and *Am*PDH3 generated with Clustal Omega (Sievers et al. 2011). The numbers on the right indicate the residue position within the protein. An asterisk (*****) indicates a single, fully conserved amino acid; a colon (**:**) indicates conservation between groups of strongly similar properties; a period (**.**) indicates conservation between groups of weakly similar properties. Residues are coloured according to their physicochemical properties, whereas the same colour indicates similar properties. For more details, the reader is referred to the frequently asked questions section of the Clustal Omega website (http://www.ebi.ac.uk/Tools/msa/clustalo/help/faq.html).

```
PDH1      AITYQHPDDLPSGVDYDFIVAGGGTAGLVVASRLSENSNWKVLVIEAGPSNKDAFVTRVP  60
PDH2      AITYQHPDDLPSGVDYDFIVAGGGTAGLVVASRLSENSKWNVLVIEAGPSNKDTPETRIP  60
PDH3      AITYQHPDDLPSDVDYDFIVAGGGTAGLVVASRLSENPDWNILVIEAGPSNKDAPETRVP  60
          ************ ************************ .*::***********:  **:*

PDH1      GLASTLGAGSPIDWNYTTIPQDGLDGRSLDYPRAKILGGCSTHNGMVYTRGSKDDWNSWA  120
PDH2      GLADNLP-GTRTDWNYTTIPQDALGGRSLNYSRAKVLGGCSTHNGMVYTRGPRDDWNYWA  119
PDH3      GLAGSLP-ASRTDWNYTTIPQDALGGRSLNYSRAKVLGGCSSHNSMVYTRGSKDDWNHWA  119
          *** .*  .:  **********.* ****:* ***:*****:**.****** :**** **

PDH1      GIIGDQGLGWDSILPAIKKAEKFTQDFTDQSVKGHIDPSVHGFDGKLSVSAAYSNISFND  180
PDH2      EITDNQALKWDNVLPIMKNTEKFSQDFLDQSMEGHIDPSVHGFDGMLSVVASYTNVSFNN  179
PDH3      DITGDQGLSWDSILPVMKKAEKFSKDFSNQSVDGHIDPSMHGHDGLLSVVSSYTNVSFND  179
           *  :*.* **.:** :*::***::** :**:.******:**.** *** ::*:*:***:

PDH1      LLFETTKELNAEFPFKLDMNDGKPIGLGWTQYTIDNHAERSSSATSYLESTGDNVHVLVN  240
PDH2      LLLETTRELSDEFPFKLDLNDGKPHGLAWTQYTIDQGAERSSSATSYLESTGDNVHVLVN  239
PDH3      LLLETTKELSDEFPFKLDLNDGNPHGLAWTQYTIDHRAERSSSATSYLESTRDNVHVLVN  239
          **:***:**. *******:***:* **.*******. ************** ********

PDH1      TLVTRVLSASGNGTDFRKVEFAVDANSPKKQLEAKKEVIVAGGVIASPQILMNSGIGERK  300
PDH2      THVTRIVS-AGNETDFRSVEFAVDANSPKKVLTAKKELILSAGVIASPQILMNSGIGGRE  298
PDH3      SRVTRIFS-AGNGTDFRSVEFAVDANSPKKVLTAKKEVILSAGVIASPQVLMNSGIGGRE  298
          : ***:.* :** ****.************ * ****:*::.*******:******* *:

PDH1      VLQAVGIDTLIDNPSVGKNLSDQGATSVMFDTTLPSTDFDVDAALTEWTNSHTGPLARGA  360
PDH2      ELQAIGVDTLIDNPSVGRNLSDQASTLLMFDTTLPNTDYDVAAALTEWDNSRSGPMAYAA  358
PDH3      ELQAIGVDTLIDNPSVGKNLSDQAATLLMFDTTLPNTDYDVAAALTEWEDSRSGPMAYGA  358
           ***:*:**********:*****.:* :*******.**:** ****** :*::**:* .*

PDH1      RLNHLTFVRLPDDKLNGQDPSSGKNSPHIEFQFAQITPQVPTLGVPKQAPLPAANSYRLL  420
PDH2      RLNHLTWVRLPDDKLSGSDPSSGHDSPHIEFQFRQISHQLPPADVPNQVQLPDPDSIGVV  418
PDH3      RLNHLTWVRLLDDKLNGSDPSSGKNSPHIEFQFMQISHQLPPADAPNQVKLPDPDSIGAV  418
          ******:*** ****.*.*****::******** **: *:*   .*:*. **  :*   :

PDH1      LQLAVVNLYSISRGSISLSDNNPFTYPLIDLNMFKEDIDIAILREGIRSAGRMFSSKAFK  480
PDH2      LQFSVVNLYSISLGSVTLNNNDPFADPIIDLNMFGDQKDIAILREGVRSARRMFSSQAFK  478
PDH3      LLLAVVNLYSVSHGSIILNDNDPFANPMIDLNMFGDQKDIAILREGVRSARRMFSSQAFK  478
          * ::******:* **: *.:*:**: *:****** :: ********:*** *****:***

PDH1      NSVNKFVYPPADATSDEDLDAFLRSSTFSYVHGVGTLSMSPKGASWGVVNPDFKVKGTSG  540
PDH2      NVVHETVYPPAGVTSDEDLDAFLRTSAVSYLHGVGTLSMSPHSASWGVVNPDFRVKGTTG  538
PDH3      DVVNGTVYPPADVTSDEDLDAFLRTSAISYWHGVGTLSMSPQNASWGVVDPDFRVKGTSG  538
          : *.  ***** .***********:*:.** **********: ******:***:****:*

PDH1      LRVVDASVIPHAPAAHTQLPVYAFAEYASALIAKSYN 577
PDH2      LRVVDASVIPRAPAGHTQIPVYTFAEHASALIADSYN 575
PDH3      LRVVDASVIPYAPAGHTQVPVYTFAEHASVLIAKSYA 575
          ********** ***.***:***:***:**.***.**
```

Fig. S2 Superimposition of the *Am*PDH1 crystal structure (PDB: 3H7U (Tan et al. 2013)) with the homology models of (**A**) *Am*PDH2 and (**B**) *Am*PDH3. All amino acids and their corresponding labels are uniformly coloured beige for *Am*PDH1, green for *Am*PDH2, and magenta for *Am*PDH3. Atom-colouring scheme for FAD: carbon (yellow), nitrogen (blue), oxygen (red), and phosphorus (orange). The figure was generated using PyMOL (http://www.pymol.org/). The homology models for *Am*PDH2 and *Am*PDH3 were generated based on the crystal structure of *Am*PDH1 as a template using the automated mode of the SWISS-MODEL workspace (http://swissmodel.expasy.org/).

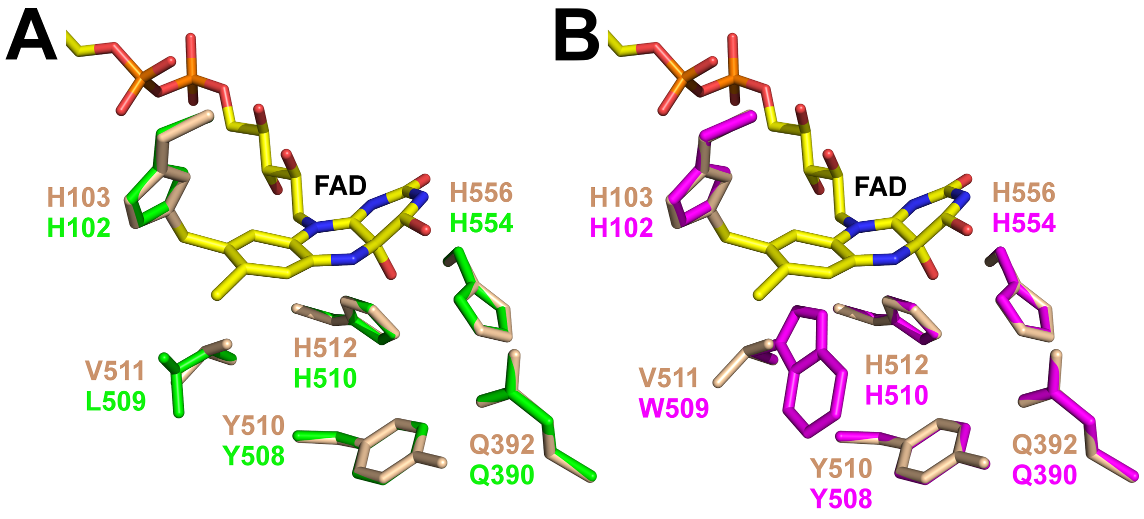

Fig. S3 UV-vis spectra of fully oxidized (solid line) and glucose-reduced (dashed line) (**A**) *Am*PDH1, (**B**) *Am*PDH2, (**C**) *Am*PDH3, and (**D**) *Am*PDH3_green. In **E** and **F**, the UV-Vis spectra of fully oxidized (**E**) *Am*PDH3_green (green solid line) and (**F**) *Am*PDH3 (yellow solid line) are compared to the spectra of the oxidized flavin domains of cellobiose dehydrogenase (CDH) from *P. chrysosporium* (dotted line) and *H. insolens* (solid line). The UV-Vis spectra of CDH were taken from an article of Igarashi and co-workers (Igarashi et al. 1999), where the authors point out that the flavin domain of *H. insolens* CDH (**E**) had a green colour and absorption maxima at 343 and 426 nm, resembling the spectrum of 6-hydroxyflavin.

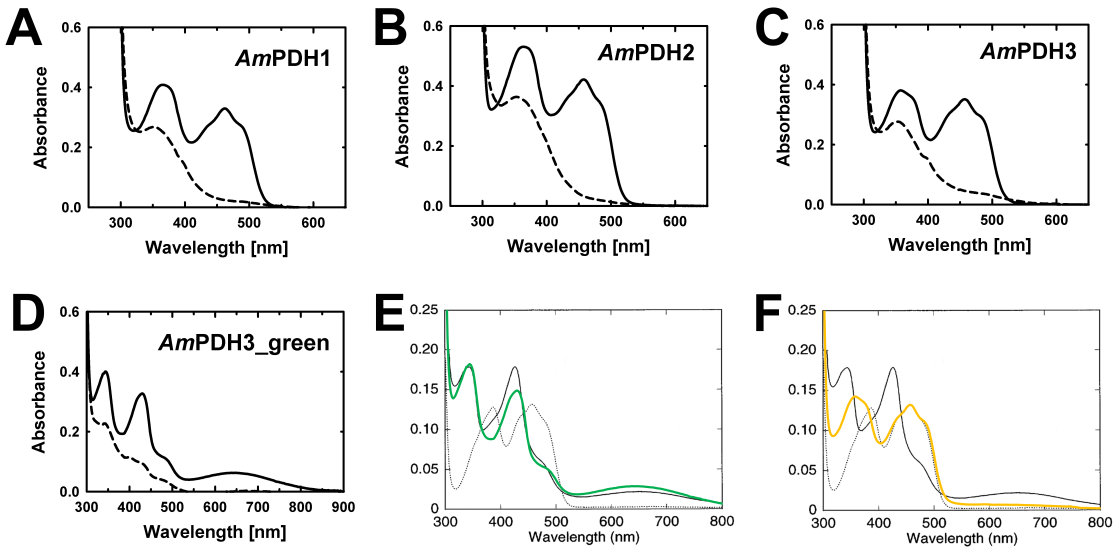

References

Graf MM, Sucharitakul J, Bren U, Chu DB, Koellensperger G, Hann S, Furtmüller PG, Obinger C, Peterbauer CK, Oostenbrink C, Chaiyen P, Haltrich D (2015) Reaction of pyranose dehydrogenase from *Agaricus meleagris* with its carbohydrate substrates. FEBS J 282:4218-4241

Igarashi K, Verhagen MF, Samejima M, Schulein M, Eriksson KE, Nishino T (1999) Cellobiose dehydrogenase from the fungi *Phanerochaete chrysosporium* and *Humicola insolens*. A flavohemoprotein from *Humicola insolens* contains 6-hydroxy-FAD as the dominant active cofactor. J Biol Chem 274:3338-3344

Sievers F, Wilm A, Dineen D, Gibson TJ, Karplus K, Li W, Lopez R, McWilliam H, Remmert M, Soding J, Thompson JD, Higgins DG (2011) Fast, scalable generation of high-quality protein multiple sequence alignments using Clustal Omega. Mol Syst Biol 7:539

Tan TC, Spadiut O, Wongnate T, Sucharitakul J, Krondorfer I, Sygmund C, Haltrich D, Chaiyen P, Peterbauer CK, Divne C (2013) The 1.6 Å crystal structure of pyranose dehydrogenase from *Agaricus meleagris* rationalizes substrate specificity and reveals a flavin intermediate. PLoS One 8:e53567
